# Optimized Expression and Characterization of a Novel Fully Human Bispecific Single-Chain Diabody Targeting Vascular Endothelial Growth Factor165 and Programmed Death-1 in *Pichia pastoris* and Evaluation of Antitumor Activity In Vivo

**DOI:** 10.3390/ijms19102900

**Published:** 2018-09-25

**Authors:** Chenghao Xiong, Yingqing Mao, Tao Wu, Nannan Kang, Mingjun Zhao, Rongrong Di, Xiaoping Li, Xuemei Ji, Yu Liu

**Affiliations:** 1 State Key Laboratory of Natural Medicines, School of Life Science and Technology, China Pharmaceutical University, Nanjing 211198, China; chenghaoxiong_cpu@163.com (C.X.); IKatayoseRyota@163.com (Y.M.); iioowt0212@163.com (T.W.); Kangnannan@126.com (N.K.); zmjun91@126.com (M.Z.); dirongcpu@163.com (R.D.); 2 Hainan Institute of Drug Research, Haikou 570311, China

**Keywords:** bispecific single-chain diabody, vascular endothelial growth factor165, programmed death-1, anti-angiogenesis, immunotherapy, *Pichia pastoris*

## Abstract

Bispecific antibodies, which can bind to two different epitopes on the same or different antigens simultaneously, have recently emerged as attractive candidates for study in various diseases. Our present study successfully constructs and expresses a fully human, bispecific, single-chain diabody (BsDb) that can bind to vascular endothelial growth factor 165 (VEGF165) and programmed death-1 (PD-1) in *Pichia pastoris*. Under the optimal expression conditions (methanol concentration, 1%; pH, 4.0; inoculum density, OD600 = 4, and the induction time, 96 h), the maximum production level of this BsDb is achieved at approximately 20 mg/L. The recombinant BsDb is purified in one step using nickel-nitrilotriacetic acid (Ni-NTA) column chromatography with a purity of more than 95%. Indirect enzyme-linked immune sorbent assay (ELISA) and sandwich ELISA analyses show that purified BsDb can bind specifically to VEGF165 and PD-1 simultaneously with affinities of 124.78 nM and 25.07 nM, respectively. Additionally, the BsDb not only effectively inhibits VEGF165-stimulated proliferation, migration, and tube formation in primary human umbilical vein endothelial cells (HUVECs), but also significantly improves proliferation and INF-γ production of activated T cells by blocking PD-1/PD-L1 co-stimulation. Furthermore, the BsDb displays potent antitumor activity in mice bearing HT29 xenograft tumors by inhibiting tumor angiogenesis and activating immune responses in the tumor microenvironment. Based on these results, we have prepared a potential bispecific antibody drug that can co-target both VEGF165 and PD-1 for the first time. This work provides a stable foundation for the development of new strategies by the combination of an angiogenesis inhibition and immune checkpoint blockade for cancer therapy.

## 1. Introduction

The development of antibody engineering has provided opportunities to generate second generation antibody drugs, including bispecific antibodies (BsAbs). Bispecific antibodies, which combine specificities of two antibodies and effectively bind to two different epitopes on the same or different antigens, are widely studied for diagnostic and therapeutic application in various diseases [1]. Blinatumomab, a bispecific antibody fragment targeting CD3 and CD19, was approved in December 2014 by the U.S. Food and Drug Administration (FDA) for the treatment of patients with Philadelphia chromosome-negative precursor B cell acute lymphoblastic leukemia (ALL) [2]. To date, at least 61 bispecific antibodies produced by 24 different bispecific antibody formats are in clinical trials and two-thirds of them have been researched and developed for cancer treatment [3]. Therefore, bispecific antibodies have many potential applications in cancer treatment as well as other refractoriness diseases.

Neovascularization and the immune responses are hallmarks of cancer and play crucial roles in the progression and metastasis of tumor cells. Vascular endothelial growth factor A (VEGF-A), a key angiogenic regulator, can promote angiogenesis through proliferation, migration, and the survival of endothelial cells [4]. Antiangiogenic therapy targeting VEGF-A/VEGF receptors’ (VEGFR) signaling pathways has become a well-established approach in oncotherapy. Bevacizumab, a recombinant humanized anti-VEGF-A monoclonal antibody (mAb), was approved by the FDA to treat metastatic colorectal cancer in combination with standard chemotherapy in 2004. Currently, it is widely used to treat various cancers, including metastatic non-small-cell lung cancer (NSCLC), metastatic renal cell carcinoma (RCC), breast cancer, epithelial ovarian cancer, and glioblastoma [5]. However, the beneficial effects of antiangiogenic monotherapy to control tumor progression lack satisfactory sustainability [6], and the resistance mediated by the adaptive and intrinsic mechanisms in anti-angiogenic therapy is a major obstacle for cancer treatment [7]. Additionally, immunotherapy has emerged as a paradigm to suppress tumor progression by initiating the immune responses and subverting cancer immune evasion in recent years. Programmed death-1 (PD-1) is an immunoglobulin superfamily member, mainly expressed by T cells, that negatively regulates human immune responses [8]. It is noteworthy that the PD-1-targeting mAb, nivolumab, has received the FDA approval for treating NSCLC, melanoma, and RCC [9] and, recently, the FDA also expanded the nivolumab for metastatic NSCLC [10]. However, it must be reported that the administration of immune checkpoint inhibitors induced immune-related adverse events such as organic inflammation and persistent high fever [11,12].

Accumulating studies have shown that antiangiogenic therapy combined with an immune checkpoint blockade can significantly increase suppressive capacity to various cancers [13,14,15]. A PD-1 blockade can sensitize tumors to antiangiogenic therapy and prolong its efficacy in metastatic breast cancer, pancreatic neuroendocrine tumors, and melanoma models and, conversely, antiangiogenic therapy can improve immune checkpoint inhibitor treatment due to the formation of intratumoral high endothelial venules that facilitate cytotoxic T cell (CTL) infiltration, activation, and tumor eradication [16,17]. Therefore, there is a bidirectional link and synergistic effect between antiangiogenic agents and immunotherapy and the development of new therapeutic agents that can inhibit angiogenesis and block the immune checkpoint simultaneously, which is a more reasonable and effective method for cancer therapy.

Previously, our laboratory obtained a fully human anti-VEGF165 mAb with low immunogenicity and great safety from five-feature transgenic mice expressing human immunoglobulin loci [18]. The anti-VEGF165 mAb can not only significantly inhibit VEGF165-stimulated proliferation of human microvascular endothelial cells (HMVECs), but also effectively target tumor tissue over-expressing VEGF in vivo [19]. During the present study, we generated a non-immunogenic, bispecific, single-chain diabody (BsDb) targeting both VEGF165 and PD-1, which serves as a potential agent for cancer therapy. To improve production levels of recombinant BsDb, we have selected a high-expressing transformant and investigated the effects of cultivation factors like methanol induction time, methanol concentration, pH value, and cell density. Furthermore, biological activities and antitumor activity of this BsDb are analyzed in vitro and in vivo.

## 2. Results

### 2.1. Construction and Transformation of the pPICZαA-Bispecific Single-Chain Diabody (BsDb) Expression Vector

**Figure 1:**
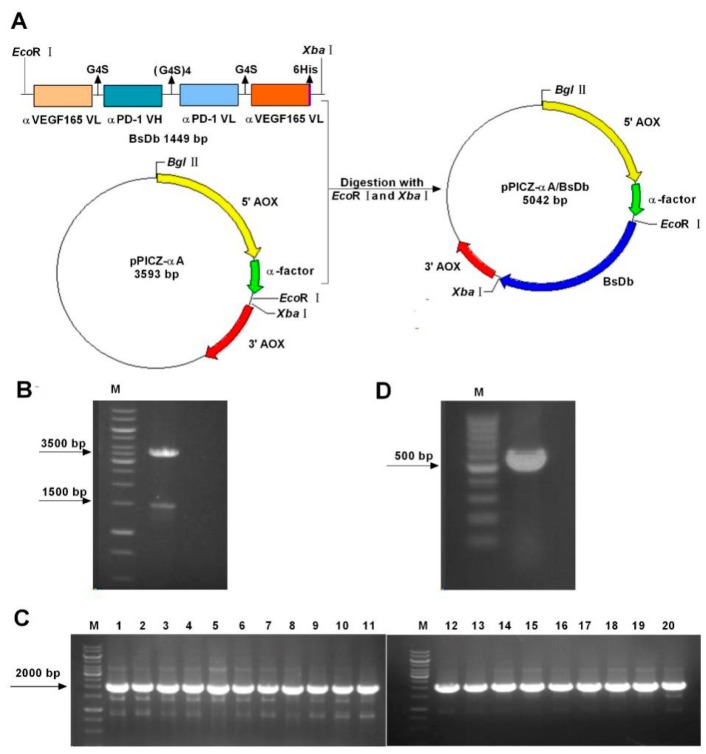
Construction and transformation of the pPICZ-αA/BsDb expression plasmid. (**A**) Schematic diagram of the pPICZ-αA/BsDb expression plasmid generation. The gene fragment of BsDb containing a 6His-tag at the C-terminal end was cloned into pPICZαA by using EcoRI/XbaI digestion; (**B**) Restriction enzyme digestion of recombinant pPICZαA-BsDb expression vector. Lane M, 1 k bp marker (Thermo, Waltham, MA, USA); (**C**) Colony PCR analysis of 20 positive recombinants obtained from YPD plates containing zeocin. Lane M, 1 k bp marker (Thermo, Waltham, MA, USA); (**D**) Colony PCR analysis of a negative clone transformed pPICZαA empty vector. Lane M, 100 bp marker (Thermo, Waltham, MA, USA).

The gene fragment of this recombinant BsDb was codon-optimized based on the *Pichia* codon-usage and synthesized by Genewiz. (Soochow, China). Sequencing data of codon-optimized BsDb was submitted to the National Center for Biotechnology Information (NCBI) nucleotide database (accession number: MH748526). The amino acid sequence and optimized nucleotide sequence, as well as GC content of this BsDb gene fragment, are represented in Figure S1. To generate the pPICZαA-BsDb expression vector, the gene fragment of BsDb with a 6His-tag at the C-terminal end was cloned into pPICZαA by using EcoRI/XbaI digestion (Figure 1A), and subsequently transformed into DH5-α cells and selected on low-salt luria-bertani (LB) plates. Recombinant positive plasmid was identified by restriction enzyme digestion with EcoRI and XbaI, which produced two DNA electrophoretic bands of ≈1500 and 3500 bp (Figure 1B). The DNA sequencing of the recombinant plasmid further confirmed that the BsDb fragment was inserted into pPICZαA correctly. The SacI linearized recombinant expression vector was transformed into *Pichia pastoris* GS115 cells by electroporation. Twenty transformants screened by yeast extract peptone dextrose medium (YPD) plates containing Zeocin were subsequently identified using PCR. The results show that pPICZαA-BsDb was successfully transferred into 20 clones (Figure 1C). The pPICZ-αA empty vector was transferred into *Pichia pastoris* and used as a negative control (Figure 1D).

### 2.2. Expression and Detection of Recombinant BsDb in Pichia pastoris

**Figure 2:**
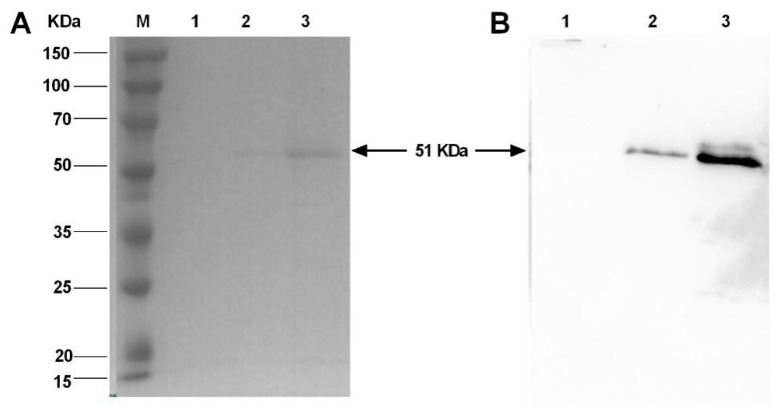
Analysis of recombinant BsDb expression in *Pichia pastoris*. (**A**) SDS-PAGE analysis: culture supernatant from a negative clone induced by 1% methanol for 96 h (lane 1); culture supernatant from the positive transformant grown under identical condition (lane 2); culture supernatant concentrated 5-fold from the positive transformant (lane 3); and protein molecular weight marker (Sangon, Shanghai, China) (Lane M); (**B**) Western blot analysis: supernatant from a negative clone (lane 1); culture supernatant from a positive transformant (lane 2); and culture supernatant concentrated 5-fold from a positive transformant (lane 3).

Following the transformation, a BsDb positive colony of *Pichia pastoris* was selected randomly and induced to express recombinant protein by methanol for 96 h. GS115 cells transformed with the empty pPICZαA vector were used as a negative control. Recombinant protein secreted in the supernatant was analyzed by Coomassie brilliant blue staining and Western blotting (Figure 2). Compared with the empty vector (Figure 2A lane 1), an expected protein band around 50 kDa was detected in the culture medium (Figure 2A lane 2). The expected protein band was observed more clearly when the supernatants were concentrated about five-fold by ultrafiltration (Figure 2A lane 3). Based on the amino acids sequence, the calculated molecular weight of BsDb was approximately 51 kDa, which was similar to the result of the SDS-PAGE measurement. Meanwhile, the recombinant protein was further analyzed using Western blotting with a mouse anti-6His-tag antibody. An obvious band on a polyvinylidene fluoride (PVDF) membrane was detected in supernatant or the five-fold concentrated supernatant of the positive transformant (Figure 2B lanes 2 and 3); however, the was no similar band in the negative control (Figure 2B lane 1). Therefore, these results show that the recombinant BsDb was successfully expressed in *Pichia pastoris*, which suggests *Pichia pastoris* might be considered a suitable host for the production of bispecific antibody fragments that comprise multi-domains.

### 2.3. Optimized Expression of Recombinant BsDb

**Figure 3:**
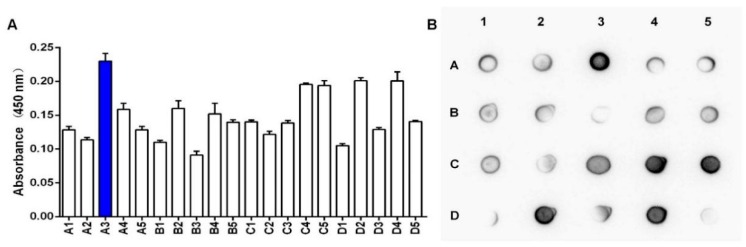
Analysis of relative expression levels of 20 positive clones. (**A**) Evaluation of the relative expression levels of various positive clones using ELISA. Recombinant BSDb relative expression levels were measured using the absorbance of 450 nm, clone A3 (blue column) possessed the highest production of the recombinant BsDb compared to the others; (**B**) Relative expression levels of BsDb in supernatants from twenty PCR positive clones were evaluated using spot Western blotting. The experiments were performed in triplicate, and the mean values ± standard deviation (SD) are presented.

To obtain high-yielding clones, we evaluated the BsDb expression level of different clones using spot Western analysis and enzyme-linked immune sorbent assay (ELISA) as described in the “Materials and Methods”. Twenty positive colonies confirmed by PCR analysis were grown in a buffered minimal glycerol-complex (BMGY) medium for 18 h and then induced in a buffered minimal methanol-complex (BMMY) medium at 30 °C for 96 h. The supernatant samples of individual clones were collected and the relative production levels of the recombinant BsDb were analyzed using ELISA and spot Western blot. The A3 colony had the highest expression of the recombinant protein compared to the others, according to the absorbance of 450 nm (Figure 3A). Simultaneously, Figure 3B also shows that clone A3 possessed high production levels of BsDb.

**Figure 4:**
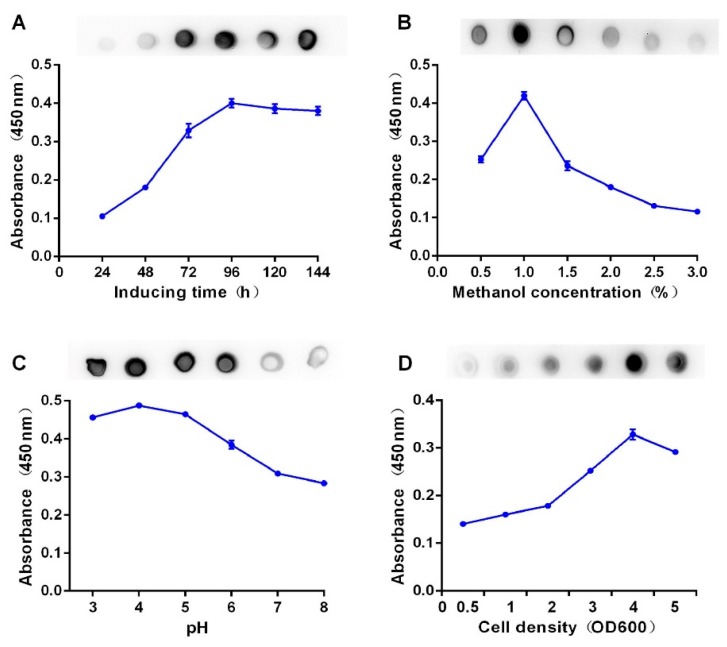
Optimization of recombinant BsDb expression in *Pichia pastoris*. Supernatants collected at each evaluated condition were analyzed using ELISA and spot Western blotting analysis. (**A**) Optimization of the methanol inducing time; (**B**) optimization of the methanol concentration; (**C**) optimization of the pH value; and (**D**) optimization of the cell density. The experiment was performed in triplicate, and the mean values ± SD were presented.

To improve the recombinant BsDb production, the optimal expression conditions of this recombinant BsDb were evaluated using spot Western analysis and ELISA. The results show that the maximum production level of this BsDb was achieved as approximately 20mg/L under the following conditions: methanol concentration, 1%; pH value, 4.0; inoculum density, OD600 = 4 and the induction time, 96 h (Figure 4).

### 2.4. Obtain the Recombinant BsDb with High Purity and High Binding Abilities Using Nickel-Nitrilotriacetic Acid (Ni-NTA) Column Chromatography

**Figure 5:**
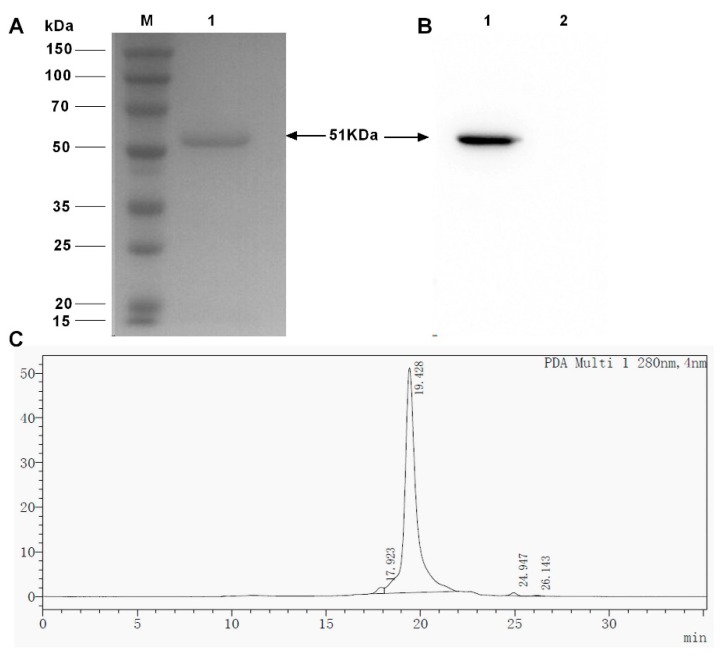
Purification of recombinant BsDb using Ni-NTA affinity chromatography. (**A**) SDS-PAGE of purified recombinant BsDb; (**B**) Western blot analysis of purified recombinant BsDb (lane 1) and 5 µg BSA was used as a negative control for Western blot analysis (lane 2), protein molecular weight marker (Sangon, Shanghai, China) (Lane M); and (**C**) HPLC analysis of purified recombinant BsDb.

Supernatants induced using methanol for 96 h under the optimal growth and induction conditions were harvested and dialyzed against a binding buffer. The expressed BsDb was purified through a nickel-nitrilotriacetic acid (Ni-NTA) agarose column due to the 6xHis-tag at the C-terminal of the recombinant protein. The purified recombinant protein was observed as a single band of about 51 kDa using sodium dodecyl sulfate polyacrylamide gel electrophoresis (SDS-PAGE) (Figure 5A) and Western blot analysis (Figure 5B). The yield of recombinant BsDb was approximately 11.4 mg/L, and the purity was more than 95%, as estimated using high performance liquid chromatography (HPLC) (Figure 5C).

**Figure 6:**
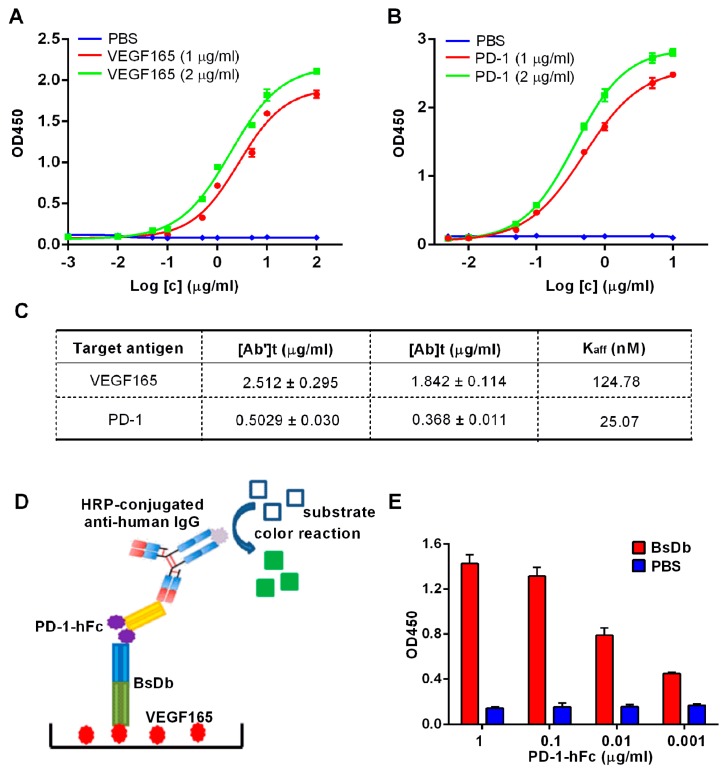
Measurement of affinities of recombinant BsDb to PD-1 and VEGF165. Binding curves of recombinant BsDb to VEGF165 (**A**) and PD-1 (**B**) tested using indirect ELISA; (**C**) The affinity constant was determined using the Beatty formula. [Ab]t and [Ab’]t represent the EC50 of high and low concentrations of antigen, respectively. PBS was used as a negative control; (**D**) Schematic diagram of sandwich ELISA; (**E**) Simultaneous binding of recombinant BsDb to both PD-1 and VEGF165 was evaluated using sandwich ELISA. PBS was used as a negative control. The experiments were performed in triplicate, and the mean values ± SD were presented.

Regarding therapeutic bispecific BsDb, high binding affinities for different antigens are essential. The affinity constants of this recombinant BsDb were assessed using indirect ELISA. The binding curves of recombinant BsDb to VEGF165 and PD-1 were represented in Figure 6A,B, respectively. Subsequent to the Beatty formula calculation [20], the results showed that the affinity constants of this BsDb for VEGF165 and PD-1 were 124.78 nM and 25.07 nM, respectively (Figure 6C). To further determine that BsDb binding to PD-1 and VEGF165 was simultaneous, another experiment, sandwich ELISA, was performed according to the schematic diagram represented in Figure 6D. The results of the sandwich ELISA demonstrated that BsDb could bind to PD-1 and VEGF simultaneously (Figure 6E). Taken together, these data suggest that the recombinant BsDb could bind to VEGF165 and PD-1 simultaneously with high binding abilities.

### 2.5. Inhibitory Activity of BsDb on Human Umbilical Vein Endothelial Cells (HUVECs) Proliferation, Migration, and Tube Formation

**Figure 7:**
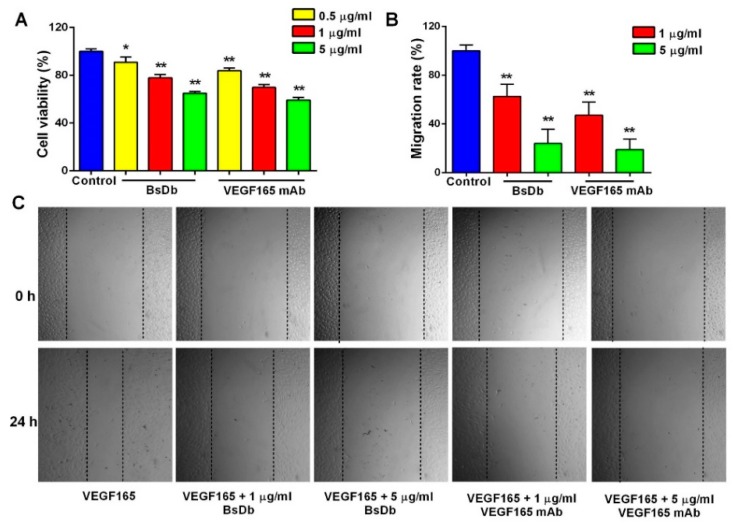
The recombinant BsDb inhibited VEGF-induced proliferation and migration of primary HUVECs. (**A**) BsDb inhibited the primary HUVECs proliferation. Primary HUVECs were cultured in 96-well plates and stimulated with 100 ng/mL VEGF165 and various concentrations of recombinant BsDb or VEGF165 mAb. Cell growth was then determined using an MTT assay; (**B**,**C**) BsDb inhibited the primary HUVECs migration. The HUVECs monolayer was scratched and placed in fresh Endothelial Cell Medium (ECM) containing 1% FBS with 100 ng/mL VEGF165 and various concentrations of recombinant BSDb or VEGF165 mAb. The HUVECs migration was photographed using a microscope after 24 h (40×) and the migration rate was calculated. The experiment was done in triplicate and the mean values ± SD were presented. * *p* < 0.05 and ** *p* < 0.01 using a two-tailed students t-test versus the control group.

Human umbilical vein endothelial cells (HUVECs) are the most commonly used cell model for VEGF-related studies since VEGF strongly stimulates the proliferation and migration of HUVEC cells [21]. Primary HUVECs were shown to express VEGFR2 at high levels (Figure S2A) using flow cytometry (FCM) analysis, which was consistent with a previously reported study [22]. Additionally, a 3-(4,5-Dimethylthiazol-2-yl)-2,5-Diphenyl Tetrazolium Bromide (MTT) assay indicated primary HUVECs proliferated intensely in response to stimulation by VEGF165 signaling (Figure S2B). To investigate the bioactivity of the BsDb to inhibit VEGF-induced angiogenesis of HUVECs in vitro, a HUVEC proliferation assay and scratch wound healing assay was performed. The results show that the recombinant BsDb displayed dose-dependent inhibitory activity on HUVEC proliferation and migration, which exhibited similar efficacy to VEGF165 mAb (Figure 7).

**Figure 8:**
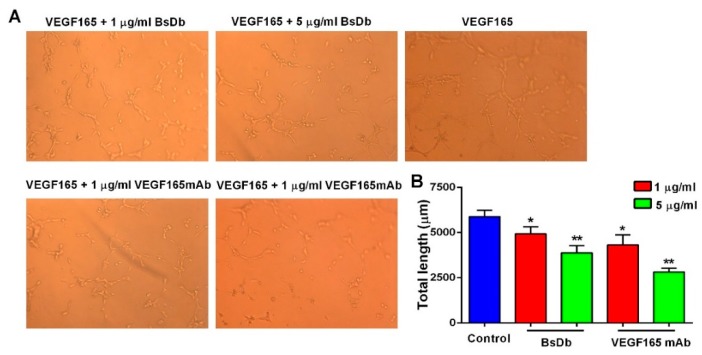
The recombinant BsDb inhibited tube formation of primary HUVECs. (**A**) HUVECs were seeded into 96-well plates that were coated with matrigel, then 100 ng/mL VEGF165 and various concentrations of BsDb or VEGF165 mAb were added to the 96-well plate, and the wells were photographed after incubation for 5 h at 37 °C (200×); (**B**) Quantification of the tube length by ImageJ software. The experiment was performed in triplicate and the mean values ± SD were shown. * *p* < 0.05 and ** *p* < 0.01 using a two-tailed students t-test versus the control group.

VEGF could promote endothelial tubular morphogenesis as a key angiogenic stimuli. To further confirm whether the recombinant BsDb was capable of inhibiting VEGF165 signaling, the HUVECs tube formation assay was used to assess the inhibitory effect of the BsDb in angiogenesis. These results showed that the recombinant BsDb and VEGF165 mAb remarkably decreased the total length of the tubes in a dose-dependent manner (Figure 8), suggesting an effective inhibitory function of the recombinant BsDb against VEGF165-stimulated tube formation in HUVECs.

### 2.6. The Recombinant BsDb Improved T Cell Proliferation and Rescued T Cells Activation

**Figure 9:**
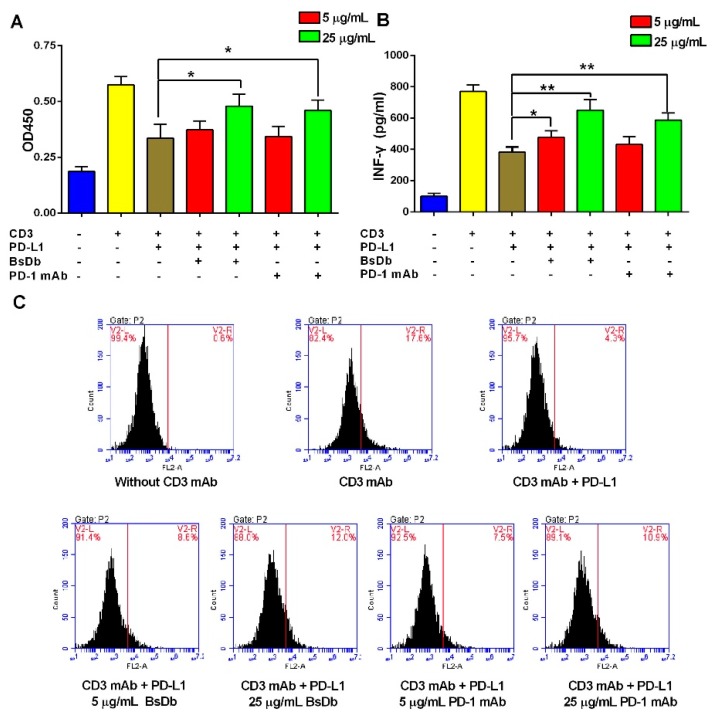
BsDb improved T cell proliferation and rescued T cell activation. (**A**) BsDb improved T cell proliferation. T cells were seeded into the 96-well plates precoated with an anti-CD3 antibody and human PD-L1. Subsequently, various concentrations of BsDb or PD-1 mAb were added into each well after incubation at 37 °C for 72 h. T cell proliferation was analyzed using the CCK-8 assay; (**B**) BsDb rescued T cell activation and increased INFγ secretion. T cells were seeded into the 96-well plates precoated with anti-CD3 antibody and human PD-L1. Subsequently, various concentrations of BsDb or PD-1 mAb were added into each well and, after incubation at 37 °C for 72 h, IFN-γ in-culture supernatants were evaluated using an ELISA kit; (**C**) BsDb improved intracellular IFN-γ of T cells using FCM analysis. The experiment was performed in triplicate and the mean values ± SD were presented. * *p* < 0.05 and ** *p* < 0.01 using a two-tailed students *t*-test versus the control group.

Flow cytometry (FCM) analysis showed that T cells were successfully isolated from peripheral blood mononuclear cells (PBMCs) with a high purity (more than 95%) using magnetic cell sorting (MACS) (Figure S3). To investigate whether the recombinant BsDb exhibited immunoactivated function on T cells, T-cell proliferation and activation assays were performed in the presence of a CD3 antibody and PD-L1 protein, according to previous studies [23]. The results showed the CD3 antibody significantly increased T cell proliferation and interferon-γ (INF-γ) release; however, these phenomena were reversed by the addition of 1 µg/mL PD-L1 protein. When the recombinant BsDb or PD-1 mAb was added, the function of T cells was reactivated, and improved proliferation and INF-γ release of T cells were observed (Figure 9A,B). Meanwhile, flow cytometry analysis showed that intracellular IFN-γ of T cells stimulated using a CD3 antibody was significantly increased, T cell activation was inhibited, and the intracellular IFN-γ level was reduced by adding PD-L1 (Figure 9C). However, BsDb and PD-1 mAb effectively increased the intracellular IFN-γ of T cells in a dose-dependent manner (Figure 9C). Taken together, the recombinant BsDb could rescue T cell activation by blocking PD-1/PD-L1 interaction.

### 2.7. The BsDb Suppressed HT29 Xenograft Tumors Growth In Vivo

**Figure 10:**
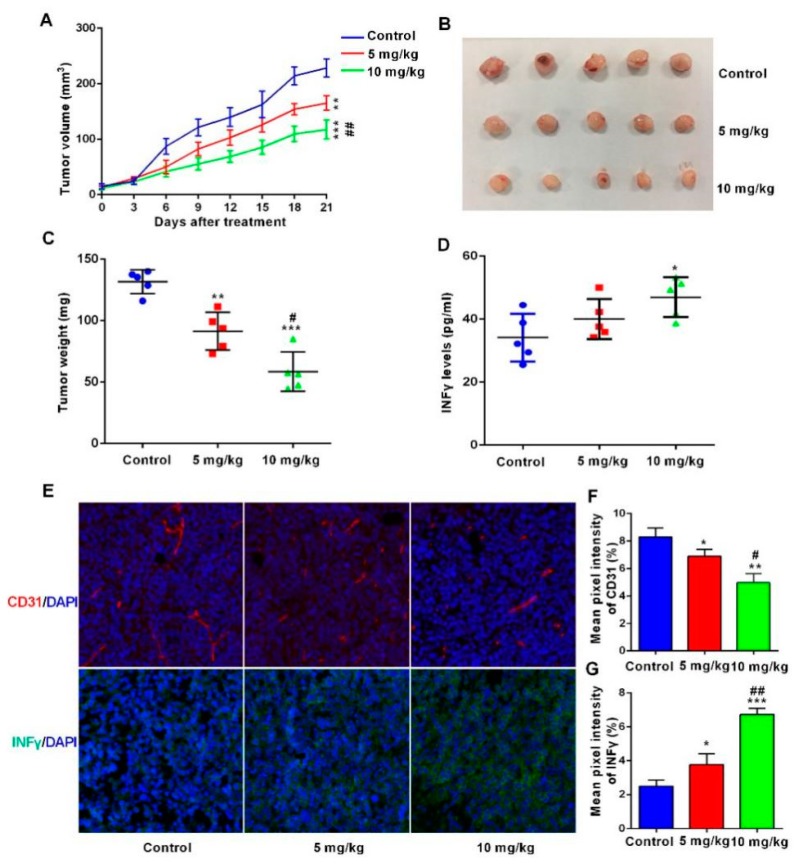
BsDb suppressed tumor growth in vivo. (**A**) The tumor volume of xenograft mice during the treatment. BsDb exerted robust antitumor activity in vivo with a dose-dependent manner; (**B**,**C**) mice were sacrificed after treatment for 21 days and tumors were collected and weighed; (**D**) Serum levels of INF-γ in different groups of mice were detected using ELISA after treatment for 21 days. Data were expressed as the mean ± SD (*n* = 5); (**E**) Immunofluorescence staining of CD31 (red fluorescence) and INF-γ (green fluorescence) in sections from tumors of different groups of mice. The cell nuclei were stained with 4′,6-diamidino-2-phenylindole (DAPI) in blue and the sections were photographed using a fluorescence microscope (200×); (**F**,**G**) quantitative analysis of CD31 and INF-γ fluorescence staining using ImageJ software. Data were expressed as the mean ± SD (*n* = 3). Statistical analysis was tested using one-way analysis variance (ANOVA) and post hoc Tukey honestly significant difference (HSD). * *p* < 0.05, ** *p* < 0.01, and *** *p* < 0.001 versus the control group; # *p* < 0.05 and ## *p* < 0.01 versus the 5 mg/kg group.

Finally, we evaluated the antitumor activity of BsDb in a xenograft mouse model. HT-29 colon carcinoma cells were subcutaneously injected into BALB/c nude mice. PBMCs were injected three times via a tail vein after 7 days, with an every-week interval, and BsDb was administered every other day for 3 weeks. The results showed that the recombinant BsDb effectively inhibited tumor development in the xenograft mouse model. The tumor volume and tumor weight of the treatment group were significantly lower than the control group (Figure 10A–C). To further verify that BsDb suppressed HT29 xenograft tumor growth by inhibiting tumor angiogenesis and activating immune responses, the authors first analyzed the serum levels of INF-γ in different groups of mice. The results showed that the serum levels of INF-γ of the high-dosage group of mice (10 mg/kg) were improved significantly compared with the control mice, illustrating that the BsDb can effectively activate immune responses in vivo (Figure 10D). Additionally, immunofluorescence staining analysis showed BsDb not only reduced the expression of CD31 in tumor tissue, but also dramatically increased INF-γ levels in the tumor microenvironment (Figure 10E–G). Taken together, these results demonstrated that the BsDb displayed potent antitumor activity in vivo by inhibiting tumor angiogenesis and activating immune responses in the tumor microenvironment.

## 3. Discussion

This study focused on the preparation of a novel, fully human bispecific antibody (BsAb), which not only inhibited tumor angiogenesis, but also activated immune responses. We first constructed BsDb co-targeting VEGF165 and PD-1 with a light-chain variable domains (VL)-to-heavy-chain variable domains (VH) orientation because some studies have reported that enhanced binding ability, affinity and in vivo biological activity were required for scFv or a diabody with a VL-to-VH orientation [24,25,26]. To express this protein, although the *E. coli* host system is considered the preferred choice for the production of recombinant proteins due to its affordability, short culture time, and high protein yield, the bioactivity of recombinant proteins expressed in *E. coli* might be lower compared to counterparts produced from *Pichia pastoris* or mammalian cells due to a lower ratio of correct folding [27]. Considering that BsDb contains four disulfide bonds, an expression system with eukaryotic protein modification of disulfide bond formation should be chosen for its production. *Pichia pastoris*, as a widely used heterologous expression system, is a distinguished host for large-scale expression of recombinant product for its efficient protein secretion, eukaryotic protein modifications, and proper protein folding capability [28]. To achieve a high-level secretion expression of the BsDb gene in *Pichia pastoris*, we optimized the gene sequence of BsDb by changing the codons to those that *Pichia pastoris* used more often. Additionally, to avoid the formation of unfavorable secondary structures, like hairpin turns, of this BsDb mRNA, we balanced the G+C content of this gene fragment. Previous studies have reported that the production levels of proteins, including biopharmaceuticals and industrial enzymes, can be significantly increased by codon-optimization for *Pichia pastoris* [29,30].

Cultivation parameters, such as induction time, cell density, methanol concentration, and culture medium pH, can affect the expression levels of recombinant proteins in the *Pichia pastoris* system. An increase in recombinant BsDb expression levels was observed in the present study before 96 h. There was a slight decrease at 120 h and 144 h, however, which was most likely due to the presence of proteases [31]. Controlling the methanol concentration was mainly to avoid the accumulation of methanol at high levels in the medium, since an excess of methanol leads to an accumulation of the toxic products of alcohol metabolism, such as formaldehyde, that can directly suppress the AOX1 enzyme activity [32]. The current research showed that the highest yield was achieved at a concentration of 1% and, when methanol concentration was increased to 1.5–3%, a decreasing trend of production was observed. The pH range of the medium can influence the production and secretion efficiency of the recombinant protein by affecting the activity of the proteases [33]. The maximum yield of this BsDb in the current study was observed at pH 4.0, and the amount of protein was decreased significantly at pH 7.0. Although high-density fermentation can increase protein production, rapid depletion of the nutrients and increased accumulation of protease can lead to the degradation of recombinant proteins and cell death when cell density is beyond the optimal range [34]. Figure 4D shows that the peak of the BsDb production appeared at OD600 ≈ 4 of cell density, and a significant decrease in protein production level was observed at higher cell densities. This might have occurred due to oxygen limitation and protease accumulation at higher cell densities.

The final yield of the recombinant BsDb reached 20mg/L by screening a high expression transformant and optimizing the cultivation conditions. Under optimal induction conditions, 5.7 mg of recombinant protein was purified from the supernatant 500 mL supernatants in one step using Ni-NTA agarose chromatography columns with a purity of more than 95%, which was consistent with other antibody fragments expressed by *Pichia pastoris* [29]. An important feature of antibodies, affinity, represents the binding capacity between an antibody and its target antigen. The results of the sandwich ELISA demonstrated BsDb can bind to PD-1 and VEGF165 simultaneously, and the *K_aff_* values of binding reactions detected by indirect ELISA between the BsDb and VEGF165 and the BsDb and PD-1 were 124.78 nM and 25.07 nM, respectively, illustrating that the BsDb could bind strongly to VEGF165 and PD-1 simultaneously, which was favorably in agreement with other reported antibody fragments [35].

To evaluate the potential therapeutic properties of the recombinant BsDb in vitro, we first identified the anti-angiogenic activity of the recombinant BsDb using primary HUVECs. The results demonstrated that the recombinant BsDb can effectively inhibit VEGF165-stimulated proliferation, migration, and tube formation in HUVECs. Compared to the full-length antibody, the recombinant BsDb had a slightly lower binding affinity to VEGF due to the monovalent binding. VEGF is a homodimer, which comprises two anti-parallel monomers linked by two disulfide bonds. The high affinity of VEGF antagonists could be achieved by multivalent binding to VEGF [36]. The high affinity between the VEGF165 and recombinant BsDb is essential to prevent free VEGF165 from binding to VEGFRs, as a therapeutic antibody drug; therefore, affinity maturation in vitro based on computational design would be used to improve the BsDb’s affinity to VEGF165.

PD-1 can suppress T cell activation when bound to its primary ligands, PD-L1, which is broadly expressed on T cells, B cells, dendritic cells, and many different tumor cells. To assess the immunoactivated function of BsDb on T cells by blocking PD-1/PD-L1 costimulation, a T-cell proliferation and activation assay was performed. Cytokines, indispensable to the proliferation and survival of T cells, such as INF-γ and IL-2, are elevated significantly when T cells are stimulated by the CD3 antibody, and PD-L1/PD-1 interaction can decrease proliferation and cytokine release of activated T cells by activation of immunosuppressed-related signaling pathways [23,37,38]. The current study also showed that INF-γ release, as well as T cell proliferation, were inhibited in activated T cells by adding recombination PD-L1 due to PD-1/PD-L1 interaction; however, the BsDb can significantly rescue PD-L1/PD-1-mediated T cell suppression and significantly improve T cell proliferation and INF-γ production in vitro.

Although both a syngeneic tumor model and an allogenetic tumor model can be used to assess immune checkpoint inhibitor activity, for the development of a therapeutic antibody drug, it is more convincing to evaluate the antitumor activity of immune checkpoint inhibitors using an immunodeficient mice model for human cancer. Additionally, a syngeneic tumor model might compromise the efficacy of immune checkpoint inhibitors due to low homologies between human PD-1 and mouse PD-1; therefore, we evaluated the BsDb’s antitumor activity in vivo using a PD-L1 positive HT29 colon cancer xenograft mouse model based on many previous studies [39,40,41]. VEGF antibody has prolonged the survival of patients with colon cancer when in combination with chemotherapy; however, clinical observations revealed the resistance to anti-angiogenic therapy in colorectal cancer was caused by various intrinsic or acquired mechanisms, including the immunosuppressive tumor microenvironment [42]. The results of the current study demonstrated that BsDb significantly inhibited the HT-29 colon carcinoma growth in vivo and reduced the tumor volume and tumor weight by approximately 50.24% and 57.48%, respectively, compared to the control group. Moreover, the angiogenesis-related protein, CD31, was decreased obviously in tumor tissue. Previous studies reported that the inhibition rate of bevacizumab on HT-29 colon carcinoma was approximately 30% in a xenograft BALB/c nude mouse model, and monotherapy of PD-1 antibody did not intensively inhibit tumor development in a mouse model of subcutaneous CT-26 colon carcinoma [22,43]. Anti-angiogenesis treatment not only resulted in a decrease of tumor microvessels, but also induced normalization of tumor blood vessels, restoring blood flow, and improved the ability to transport oxygen and cytokines, as well as small molecule drugs to the tumor microenvironment [44]. Further investigation showed that BsDb activated the immune responses and increased serum levels of INF-γ in vivo. Additionally, in treatment group mice, especially for the 10 mg/kg group, a significant increase of INF-γ levels was observed in the tumor microenvironment, which might be based on the delivery of normalized tumor blood vessels. Taken together, this study’s BsDb might possess a synergistic effect to overcome drug resistance during cancer therapy, compared to monotherapy, and could be considered as a potential candidate for cancer therapy.

To summarize, the present study has successfully prepared a fully human BsDb, targeting both VEGF165 and PD-1 in *Pichia pastoris*, which possessed high affinities and high activities, as well as robust antitumor activity in vitro and in vivo. Preliminary mechanisms responsible for its efficacy included tumor angiogenesis inhibition and immune response activation. Nevertheless, further studies will be necessary to address, in depth, the BsDb’s mechanisms as well as compare the efficacy of BsDb with full-length VEGF165 or PD-1 mAb in other xenograft models. Additionally, the small size of the bispecific antibody fragments leads to rather rapid renal elimination in vivo; therefore, strategies including conjugation of polyethylene glycol (PEG) or introduction of disulfide bonds will be applied to extend the half-life of BsDb [41,45]. Based on the high efficiency and low toxicity of antibody drugs, this study’s BsDb might provide a new, more effective, and safe strategy by combining angiogenesis inhibitors and immune checkpoint blockades for cancer therapy.

## 4. Materials and Methods

### 4.1. Strains and Plasmids

*E. coli* DH5α, as a host for DNA replication, was cultured in Luria-Bertani (LB) medium supplemented with 100 µg/mL ampicillin. A low-salt (0.5% sodium chloride) LB medium was used for zeocin selection of transformants, and antibiotics concentration was 100 µg/mL ampicillin and 25 µg/mL zeocin. *Pastoris*-strain GS115 was cultured in a yeast extract peptone dextrose (YPD) medium, and YPD plates containing different levels of zeocin (100, 500, and 1000 µg/mL) were used for the selection of positive *Pichia pastoris* transformants. To investigate protein expression, a recombinant *Pichia pastoris* colony was grown in a buffered minimal glycerol-complex medium (BMGY), and then inoculated into a fresh buffered minimal methanol-complex medium (BMMY). The pPICZαA expression plasmid (Invirtrogen, Carlsbad, CA, USA) was used as a vector for the expression of this BsDb in *Pichia pastoris*.

### 4.2. Cell Culture

HT-29 colon carcinoma cells were purchased from American Type Culture Collection (ATCC) and cultured in Dulbecco’s Modified Eagle Medium (DMEM) containing 10% (*v*/*v*) fetal bovine serum (FBS) at 37 °C in a 5% (*v/v*) CO_2_ incubator. Primary human umbilical vein vascular endothelial cells (HUVECs), kindly provided by Professor Li Jing (State Key Laboratory of Reproductive Medicine, Nanjing Medical University, China), and cultured in ECM complete medium (ScienCell, San Diego, CA, USA) at 37 °C in a 5% (*v*/*v*) CO_2_ incubator. PBMCs were isolated from leukapheresis products, kindly provided by the Nanjing Red Cross Blood Center (Nanjing, China).

### 4.3. Construction of the BsDb Expression Vector

The BsDb gene fragment was designed based on the amino acid sequences of the heavy-chain variable domains (VH) and light-chain variable domains (VL) of the fully human VEGF165 mAb [18] and PD-1mAb (nivolumab, BMS, New York, NY, USA) [46]. To facilitate purification and detection of this BsDb, an additional sequence encoding of a 6× His tag was introduced at the 3′ end of the gene fragment. The BsDb gene fragment was amplified by primer 1 and primer 2 (Table S1), and inserted into the pPICZαA to generate the pPICZαA-BsDb expression vector. The recombinant expression vector was transformed into DH5-α cells, selected on low-salt LB plates, and confirmed using colony PCR. To further confirm that the BsDb gene fragment was correctly inserted into the plasmid, the pPICZαA-BsDb recombinant expression vector was digested by EcoR I/Xba I enzymes and sequenced.

### 4.4. Transformation of Pichia pastoris and Selection of Recombinant Clones

The pPICZαA-BsDb recombinant plasmid was linearized with SacI and then introduced into competent cells of the *Pichia pastoris* strain, GS115, by MicroPulser™ (Bio-Rad, conditions: 2.2 kV, 6 ms). Transformants were placed in ice-cold 1 M sorbitol, regenerated at 30 °C for 2 h, and then plated on YPD medium plates containing 100 µg/mL zeocin and grown at 30 °C for 3 days. Recombinant clones purified from YPD plates were further selected with higher zeocin concentrations and analyzed using colony PCR with AOX1-F and AOX1-R primers (Table S1). The empty pPICZαA vector was used as a negative control and transformed into *Pichia pastoris*.

### 4.5. Expression and Selection of High-Expressing Clones

One randomly selected monoclonal transformant and one negative transformant were individually cultured in 10 mL BMGY at 30 °C with agitation at 250 rpm for 18 h, and then the cells were collected using centrifugation and gently resuspended in BMMY medium to induce expression of recombinant proteins. Methanol was added every 24 h to a final concentration of 1% (*v*/*v*) to maintain induction for 96 h. Following induction, supernatants were collected and analyzed using SDS-PAGE and Western blotting.

To obtain high-expressing clones, supernatants of twenty positive clones were collected and spotted directly onto the PVDF membrane (Thermo, Waltham, MA, USA) and an anti-6× His HRP-conjugated antibody (Sangon, Shanghai, China) was used as a probe to detect the expression level of the BsDb. Meanwhile, ELISA was used as another method to further verify the expression of different clones. Briefly, individual wells of ELISA plates were coated with 50 µL of supernatants, which had been diluted with 50 µL coating buffer (15 mM Na_2_CO_3_, 35 mM NaHCO_3_, pH 9.6) overnight at 4 °C. Following washing, ELISA plates were blocked with 3% BSA in phosphate buffered solution with Tween (PBST) (136.9 mM NaCl, 1.5 mM KH_2_PO_4_, 2.5 mM Na_2_HPO_4_·12H_2_O, 2.7 mM KCl, and 0.5% Tween-20) and were incubated for 2 h at 37 °C. Following several washes, HRP-conjugated an anti-his-tag antibody, which had been diluted with 1% BSA in PBST (1:3000), was added to each well and incubated for 1 h. The expression level of BsDb was reflected by the absorbance values at 450 nm.

### 4.6. Optimization of the Conditions for the Recombinant BsDb Expression

To achieve high production levels of this BsDb, the culture conditions including induction time, methanol concentration, pH value, and inoculum density were optimized. All culture conditions were maintained at constant levels except for the one being monitored. The supernatant from each experiment was collected and analyzed using spot Western analysis and ELISA. To determine the induction time, supernatant samples were collected from 24 h to 144 h, with 24 h intervals. To investigate the optimal methanol concentration, various concentrations of methanol (0.5, 1.0, 1.5, 2.0, 2.5, and 3.0% *v*/*v*) were used to induce protein expression. To optimize the pH value, the pH values (3.0, 4.0, 5.0, 6.0, 7.0, and 8.0) of the BMMY medium were adjusted with a 100 mM potassium phosphate buffer. The production of recombinant protein in the supernatant was evaluated after 96 h of induction. To obtain the optimal inoculum concentration of protein expression, a high-expressing transformant was inoculated into a fresh BMMY medium at six different cell densities (OD600 = 0.5, 1, 2, 3, 4, and 5).

### 4.7. Purification of the Recombinant BsDb

Following induction using methanol for 96 h, the supernatant of the high-expressing clone was collected and dialyzed against a binding buffer (20 mM phosphate buffer, 300 mM NaCl, pH 7.4) overnight using a 10 kDa cutoff dialysis membrane (Millipore, Billerica, MA, USA). Subsequent to filtering through 0.22 um filters (Millipore, Billerica, MA, USA), the supernatants were added to a nickel-nitrilotriacetic acid (Ni-NTA) agarose column (GE, Boston, FA, USA) with 1 mL/min flow velocity, and then the column was washed with ten column volumes of binding buffer. The interest protein bound to the column was eluted with 250 mM imidazole in a binding buffer. The elution fraction containing the interest protein was dialyzed against PBS overnight to remove the imidazole. Finally, the protein was concentrated using ultrafiltration and the concentration of the purified protein was measured using a BCA protein quantification kit (CW biotech, Beijing, China) and HPLC (Shimadzu, Tyoto, Japan) was used to determine the purity of this BsDb, as described in our previous study [47].

### 4.8. SDS-PAGE and Western Blot

Supernatant samples and purified recombinant protein were analyzed using electrophoresis on a 12% separation gel. Protein bands on gels were visualized using Coomassie Brilliant Blue staining. To perform the Western blot analysis, protein samples were transferred onto a PVDF membrane using a wet transfer system (Bio-Rad, Hercules, CA, USA) and then incubated with a 1:2000 dilution of mouse HRP-conjugated anti-6× His antibody in tris-buffered saline with Tween (TBST) (150 mM NaCl, 20 mM Tris, 0.5% Tween-20, PH 8.0) containing 3% skim milk. Finally, protein bands were detected using a chromogen-based detection system (Tanon, Shanghai, China) after washing three times by TBST.

### 4.9. Measurement of Affinity by Indirect ELISA

The affinity constant of the BsDb was determined using indirect ELISA according to the following procedures [20]. Individual wells of ELISA plate were coated with 100 µL of recombinant human VEGF165 (or PD-1), which was diluted to two concentrations ([Ag’] = 1 µg/mL and [Ag] = 2 µg/mL) using a coating buffer. PBST was used as a washing buffer and 3% BSA in PBST was used as a blocking buffer. Following washing and blocking, the purified BsDb was added to the wells with different concentrations and anti-6× His HRP-conjugated antibody served as a secondary antibody. The color reaction was performed with the addition of 100 µL TMB and incubated for 20 min at room temperature in the dark. Then, 100 μL 2 M H_2_SO_4_ was added to each well to stop the color reaction. The absorbance values at 450 nm were determined with a microplate reader (Molecular Devices, Silicon Valley, CA, USA). The affinity constant (*K_aff_*) was calculated according to the Beatty formula, *K_aff_* = (*n* − 1)/2(*n*[Ab’]t − [Ab]t) where [Ab]t and [Ab’]t represent the EC50 of high and low concentrations of antigen, respectively; *n* = ([Ag’]/[Ag]). PBS was used as a negative control.

### 4.10. Sandwich ELISA

Sandwich ELISA was performed according to the following procedures. Individual wells of the ELISA plate were coated with 100 µL recombinant human VEGF165, which was diluted to 1 µg/mL with a coating buffer. PBST was used as a washing buffer and 3% BSA in PBST was used as a blocking buffer. Following washing and blocking, 100 µL purified BsDb (1 µg/mL) was added into individual wells of the ELISA plate and incubated at 37 °C for 2 h. Following washing several times, recombinant PD-1-hFc (Novoprotein, Shanghai, China) was added to each well at different concentrations, and HRP-conjugated anti-human IgG antibody (Sangon, Shanghai, China) served as a secondary antibody. The color reaction was performed with the addition of 100 µL TMB and incubated for 20 min at room temperature in the dark. Then, 100 μL 2 M H_2_SO_4_ was added to each well to stop the color reaction. The absorbance values at 450 nm were determined with a microplate reader (Molecular Devices, Silicon Valley, CA, USA).

### 4.11. HUVECs Proliferation Assay

HUVECs proliferation was observed using a methyl thiazolyl tetrazolium (MTT) assay. Briefly, the logarithm phase HUVECs were resuspended with ECM complete medium and seeded in a 96-well plate (5 × 10^3^ per well) followed by incubation for 12 h. Adhered HUVECs were serum-starved with an ECM medium containing 1% FBS and were treated with various concentrations of BsDb or VEGF165 mAb that were pre-incubated with 100 ng/mL VEGF165 for 72 h. Subsequently, 15 µL MTT was added to each well, and the plate was incubated for another 4 h. The cell supernatants then were removed and 200 µL DMSO was added to each well. Finally, the 490 nm absorbance was measured using a microplate reader (Molecular Devices, Silicon Valley, CA, USA) to determine the cell viability.

### 4.12. HUVECs Migration Assay

Effects of BsDb on the migration ability of HUVECs were evaluated using a scratch wound healing assay. Briefly, HUVECs were inoculated into 24-well plates with a density of 5 × 10^4^/well to form a monolayer. The monolayer was scratched by a sterile yellow 200 µL pipette tip and then washed three times with PBS. Cells were placed in a fresh ECM medium containing 1% FBS with various concentrations of BsDb or VEGF165 mAb that were pre-incubated with 100 ng/mL VEGF165 for 30 min at room temperature. HUVECs migration was photographed after 24 h at 40-fold magnification using an inverted microscope and the data were expressed as a percentage of the migration of the control group.

### 4.13. HUVECs Tube Formation Assay

A tube formation assay, using matrigel, was performed according to the manufacturer′s instructions (BD, Franklin Lakes, NJ, USA). Briefly, a 96-well plate was coated with 80 µL/well Matrigel. Following polymerization, the HUVECs being serum-starved overnight were resuspended in ECM with 0.5% FBS with 8 × 10^4^/mL and seeded in a 96-well plate (100 µL/well). Various concentrations of BsDb or VEGF165 mAb were premixed with VEGF165 (100 ng/mL) for 30 min and added to the 96-well plate. Following incubation for 5 h at 37 °C, the wells were photographed at 200-fold magnification in three randomized fields, and the tube length was measured using ImageJ 1.46r software (1.46r, NIH, MA, Bethesda, USA).

### 4.14. T Cell Proliferation Assay

PBMCs were isolated from leukapheresis products using Ficoll-Paque density gradient centrifugation. T cells were isolated from PBMCs by MACS according to the manufacturer′s instructions (Miltenyi, Cologne, Germany) and purified human T cells were analyzed using a CD3-FICT antibody (Sino Biological Inc., Beijing, China). T cell proliferation was analyzed using a CCK-8 assay. Briefly, T cells (5 × 10^4^ per well) were added into 96-well plates precoated with 100 µL anti-CD3 antibody (1 µg/mL) (Sino Biological Inc., Beijing, China) or 100 µL anti-CD3 antibody (1 µg/mL) + 100 µL PD-L1 (1 µg/mL) (Novoprotein, Shanghai, China). Later, various concentrations of BsDb or PD-1 mAb were added into each well and then incubated at 37 °C for 72 h. Next, 10 µL CCK-8 was added to each well, and the plate was incubated for another 6 h. Finally, T cell proliferation was reflected using absorbance values at 450 nm.

### 4.15. T Cell Activation Assay

To activate T cells, T cells (5 × 10^4^ per well) were added into 96-well plates precoated with 100 µL anti-CD3 antibody (1 µg/mL) or 100 µL anti-CD3 antibody (1 µg/mL) and 100 µL PD-L1 (1 µg/mL). Subsequently, various concentrations of BsDb or PD-1 mAb were added into each well, and then the plates were incubated at 37 °C for 72 h. IFN-γ in culture supernatants was evaluated using an ELISA kit according to the instructions (Neobioscience, Shenzhen, China). Meanwhile, to further analyze intracellular IFN-γ, T cells were fixed with 4% paraformaldehyde and permeabilized with Permeabilization Wash Buffer (Yeasen, Shanghai, China). Cells then were incubated with anti-IFN-γ-PE (Thermo, Waltham, MA, USA) and subsequently analyzed by flow cytometry (BD, city, NJ, USA).

### 4.16. Evaluation Antitumor Activity of BsDb In Vivo

Approximately 4–6-week-old female BALB/c nude mice were purchased from the Comparative Medicine Center of Yangzhou University and kept in a specific pathogen-free (SPF) condition. HT29 cells were injected subcutaneously into the right flank to establish the xenograft model. Tumor volumes were measured and calculated according to the formula V = 0.5 × (length × width^2^) every three days. When tumor volumes reached approximately 20 mm^3^, mice were randomly separated into three groups and treated intraperitoneally every other day for 3 weeks as follows: group A had 5 mg/kg BsDb; group B had 10 mg/kg BsDb; group C had sterile PBS. To provide the human immune system to BALB/c nude mice, PBMC (1 × 10^8^) were injected three times via a tail vein at every week intervals after the treatment began. Mice were sacrificed, and tumor tissue and serum were collected after three weeks of treatment. Serum levels of INF-γ were analyzed with an ELISA kit (Neobioscience, Shenzhen, China) according to the instructions. CD31 and INF-γ in tumor tissue were detected using immunofluorescence staining. Briefly, tumor tissues were fixed in 4% paraformaldehyde, embedded in paraffin, and subsequently processed into sections. The sections were incubated with anti-CD31 and anti-INF-γ antibodies (Sino Biological Inc., Beijing, China), respectively. Then, slices were washed several times using PBS and treated with Alexa Fluor 488 or PE-labeled secondary antibodies. Finally, slides were counterstained with DAPI and analyzed using a fluorescence microscope. All procedures were approved by the Animal Ethics Committee of China Pharmaceutical University (Approval No.:201801031; 23 May 2018).

### 4.17. Statistical Analysis

Data were presented as means ± standard deviation. GraphPad Prism 6.0 software (GraphPad Software, CA, USA) was used to analyze the results. Statistical analysis between two groups were tested using a 2-tailed student’s t-test. Statistical analysis between more than two groups were tested using one-way analysis of variance (ANOVA) followed by a post hoc Tukey’s test. *p* < 0.05 and *p* < 0.01 were considered to be statistically significant and highly significant, respectively.

## Supplementary Materials

Supplementary materials can be found at http://www.mdpi.com/1422-0067/19/10/2900/s1.

## Abbreviations

BsDb	Bispecific single-chain diabody
BsAb	Bispecific antibody
VEGF165	Vascular endothelial growth factor165
PD-1	Programmed death-1
HUVECs	Human umbilical vein endothelial cells
PBMCs	Peripheral blood mononuclear cells
mAb	mAb
MTT	Methyl thiazolyl tetrazolium
ELISA	Enzyme-linked immune sorbent assay
SDS-PAGE	Sodium dodecyl sulfate polyacrylamide gel electrophoresis
HPLC	High performance liquid chromatography
MACS	Magnetic cell sorting
FCM	Flow cytometry
YPD	Yeast extract peptone dextrose medium
BMGY	Buffered minimal glycerol-complex medium
BMMY	Buffered minimal methanol-complex medium
