# Supplementary material for: Optimized Expression and Characterization of a Novel Fully Human Bispecific Single-Chain Diabody Targeting Vascular Endothelial Growth Factor165 and Programmed Death-1 in Pichia pastoris and Evaluation of Antitumor Activity In Vivo
Source: Int J Mol Sci. 2018 Sep 25;19(10):2900. doi: 10.3390/ijms19102900 (PMC6213929; doi:10.3390/ijms19102900)
Supplement: Supplementary file 1 [file ijms-19-02900-s001.zip › ijms-353470-supplementary.pdf]

Optimized Expression and Characterization of a Novel Fully Human Bispecific Single-Chain Diabody Targeting Vascular Endothelial Growth Factor165 and Programmed Death-1 in *Pichia pastoris* and Evaluation of Antitumor Activity In Vivo

Chenghao Xiong, Yingqing Mao, Tao Wu, Nannan Kang, Mingjun Zhao, Rongrong Di, Xiaoping Li, Xuemei Ji and Yu Liu

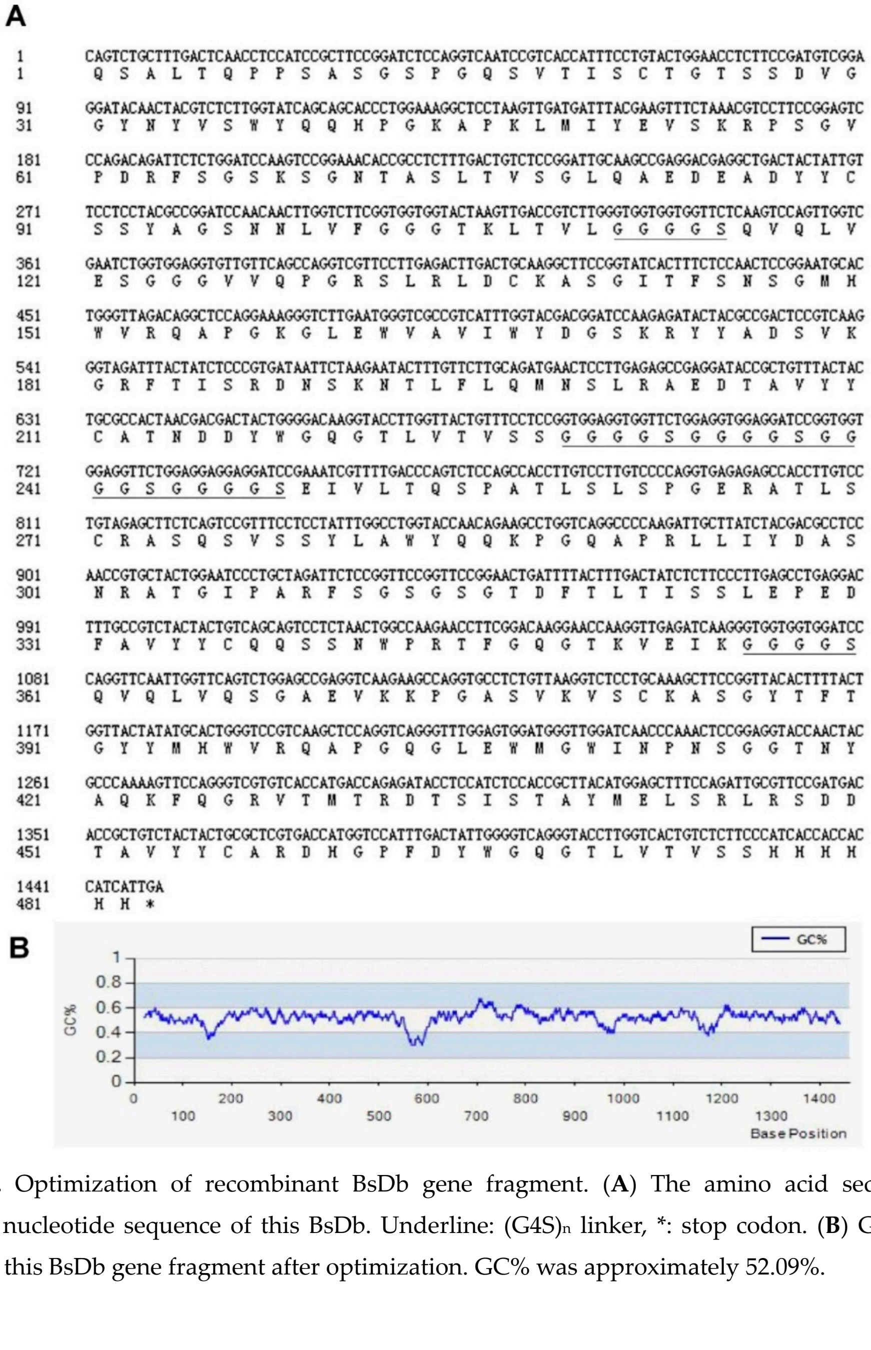

Figure S1. Optimization of recombinant BsDb gene fragment. (**A**) The amino acid sequence and optimized nucleotide sequence of this BsDb. Underline: (G4S)n linker, *: stop codon. (**B**) G+C content analysis of this BsDb gene fragment after optimization. GC% was approximately 52.09%.

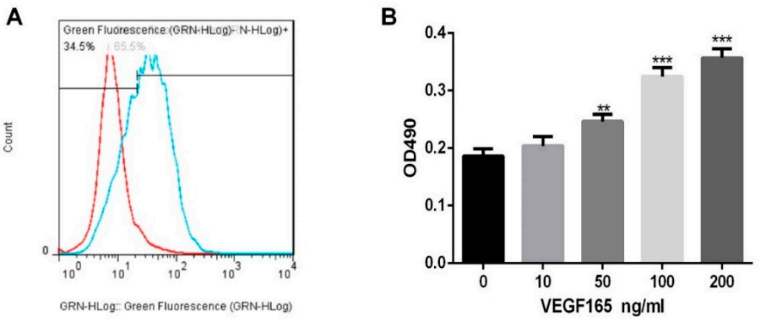

Figure S2. Identification of primary HUVECs. (**A**) VEGFR2 expression on primary HUVECs was detected using mouse anti-human VEGFR2 antibody (Sino Biological Inc., Beijing, China) and FITC-conjugated Secondary antibody (CW biotech, Beijing, China) by FCM. FCM analysis indicated VEGFR2 expressed on primary HUVECs with high levels. (**B**) VEGF165-induced proliferation of primary HUVECs was detected by MTT assay. The experiment was done in triplicate, and the mean values ± SD were presented. **p* < 0.05, ** *p* < 0.01, two-tailed students t-test, versus control group.

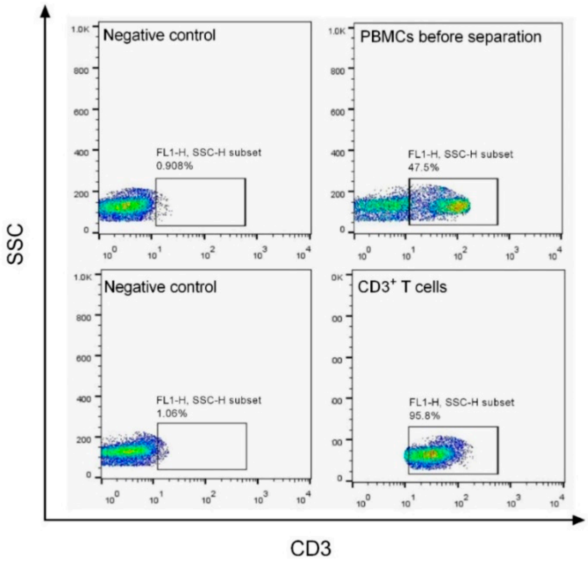

Figure S3. Analysis of T cells isolated from PBMCs. T cells were isolated from PBMCs using CD3 microbeads (Miltenyi, Cologne, Germany) by MACS. Purity of T cells was analyzed by FCM using a FITC-conjugated CD3 antibody (Sino Biological Inc., Beijing, China). Purity of T cells before and after separation were approximately 47.5% and 95.8%, respectively.

Table S1. Primers for PCR amplification of recombinant BsDb and colony PCR.

primer 1	5′-GGAATTCCAGTCTGCTTTGACTCAA-3′
primer 2	5′-GCTCTAGATCAAT GATGGTGGTGGTGA-3′
AOX1-F	5′-GACTGGTTCCAATTGACAAGC-3′
AOX1-R	5′-GCAAATGGCATTCTGACATCC-3′

Underline: restriction sites.
